# Wood Specific Gravity Variations and Biomass of Central African Tree Species: The Simple Choice of the Outer Wood

**DOI:** 10.1371/journal.pone.0142146

**Published:** 2015-11-10

**Authors:** Jean-François Bastin, Adeline Fayolle, Yegor Tarelkin, Jan Van den Bulcke, Thales de Haulleville, Frederic Mortier, Hans Beeckman, Joris Van Acker, Adeline Serckx, Jan Bogaert, Charles De Cannière

**Affiliations:** 1 Landscape Ecology and Plant Production Systems Unit, Université libre de Bruxelles, CP264-2, B-1050 Bruxelles, Belgium; 2 BIOSE Department, Gembloux Agro-Bio Tech, Université de Liège, B-5030 Gembloux, Belgium; 3 Ecole Régionale post-universitaire d’Aménagement et de gestion Intégrés des Forêts et Territoires tropicaux, Kinshasa, DR Congo; 4 UGCT, University Ghent Centre for X-ray Tomography, Proeftuinstraat 86, 9000 Ghent, Belgium; 5 Laboratory for Wood Biology and Xylarium, Royal Museum for Central Africa, Tervuren, Belgium; 6 UPR BSEF, CIRAD, Campus International de Baillarguet, F-34398 Montpellier, France; 7 Behavioural Biology Unit, University of Liege, Liege, Belgium; 8 Conservation Biology Unit, Royal Belgian Institute of Natural Sciences, Brussels, Belgium; Chinese Academy of Forestry, CHINA

## Abstract

**Context:**

Wood specific gravity is a key element in tropical forest ecology. It integrates many aspects of tree mechanical properties and functioning and is an important predictor of tree biomass. Wood specific gravity varies widely among and within species and also within individual trees. Notably, contrasted patterns of radial variation of wood specific gravity have been demonstrated and related to regeneration guilds (light demanding vs. shade-bearing). However, although being repeatedly invoked as a potential source of error when estimating the biomass of trees, both intraspecific and radial variations remain little studied. In this study we characterized detailed pith-to-bark wood specific gravity profiles among contrasted species prominently contributing to the biomass of the forest, i.e., the dominant species, and we quantified the consequences of such variations on the biomass.

**Methods:**

Radial profiles of wood density at 8% moisture content were compiled for 14 dominant species in the Democratic Republic of Congo, adapting a unique 3D X-ray scanning technique at very high spatial resolution on core samples. Mean wood density estimates were validated by water displacement measurements. Wood density profiles were converted to wood specific gravity and linear mixed models were used to decompose the radial variance. Potential errors in biomass estimation were assessed by comparing the biomass estimated from the wood specific gravity measured from pith-to-bark profiles, from global repositories, and from partial information (outer wood or inner wood).

**Results:**

Wood specific gravity profiles from pith-to-bark presented positive, neutral and negative trends. Positive trends mainly characterized light-demanding species, increasing up to 1.8 g.cm^-3^ per meter for *Piptadeniastrum africanum*, and negative trends characterized shade-bearing species, decreasing up to 1 g.cm^-3^ per meter for *Strombosia pustulata*. The linear mixed model showed the greater part of wood specific gravity variance was explained by species only (45%) followed by a redundant part between species and regeneration guilds (36%). Despite substantial variation in wood specific gravity profiles among species and regeneration guilds, we found that values from the outer wood were strongly correlated to values from the whole profile, without any significant bias. In addition, we found that wood specific gravity from the DRYAD global repository may strongly differ depending on the species (up to 40% for *Dialium pachyphyllum*).

**Main Conclusion:**

Therefore, when estimating forest biomass in specific sites, we recommend the systematic collection of outer wood samples on dominant species. This should prevent the main errors in biomass estimations resulting from wood specific gravity and allow for the collection of new information to explore the intraspecific variation of mechanical properties of trees.

## Introduction

Recent development of international programs aiming to reduce emissions from deforestation and forest degradation in the tropics (i.e., the REDD+;[1]) drew the attention of a wide scientific community to wood density. Indeed, to calculate the carbon budget of a forest, the biomass of all the trees composing the forest is estimated through the measurement of several structural parameters (i.e., the diameter at breast height, the wood density and the total height) in allometric models[2,3]. While the main source of error in biomass estimation remains the choice of the allometric model[2–5], potential errors on wood density measurement cannot be overlooked as it is recognized to be the second best predictor of the biomass of a tree[2,3,6]. Wood density, or more conventionally the wood specific gravity (WSG), i.e., wood oven-dry mass divided by its green volume, as used in most studies developing biomass allometric models[2,3], ranges from 0.1 to 1.5 g.cm^-^³ among tropical trees[7,8]. This variation is well conserved across the world’s tropical regions[9,10]. However, most species exhibit values close to the mean[9], oscillating between 0.56 and 0.63 g.cm^-^³[8]. The wood specific gravity is strongly conserved across phylogenies[8,11], so genus averages are often used for biomass estimation when the species are not identified in the field[12]. The variation in wood specific gravity has been shown to be greater among than within species[13,14], with substantial differences between light-demanding and shade-tolerant species[8,15]. Wood specific gravity integrates many aspects of wood mechanical properties[8,16] and is consequently often used as a proxy to understand the stature and functioning of tropical tree species[8,11,17]. The fast growth of light-demanding species in the early stages is often associated with the production of soft wood with low density[18,19]. Such a trade-off between growth and density is sometimes assumed to provide a competitive advantage but reduce tree longevity[20]. Conversely, many shade-tolerant species are believed to invest in denser wood and grow more slowly, but to persist longer in the understory. This higher wood specific gravity provides greater resistance to physical damage and potential pathogens[21,22], and reduces the investment in metabolism and conduction[23,24]. As a result, the mean wood specific gravity of a forest stand is often related to the successional status, with a lower wood specific gravity in secondary than in old-growth forests reported in central and southern America[25,26], in south-eastern Asia[27], and more recently in Central Africa[28].

The wood specific gravity also presents substantial variations within a tree, both along its vertical[10,29] and its radial profile[18,26,30]. Exceptional radial variations have been reported for large tropical pioneer species where the ratio between the outer and the inner wood can reach 4.3-fold[20]. This radial increase of wood density from pith-to-bark is often related to the variation of the tree growth rate during ontogeny[9]. As light-demanding species are particularly sensitive to light exposure, it is assumed that they need to invest in a denser wood to persist in a mature forest, i.e., when the competition for light is stronger[18,20]. By contrast, shade-tolerant species tend to show a decreasing trend through ontogeny, possibly due to gradual shift from the shaded understory to increased light exposure, and the consequent increase in metabolic activity[19]. Non-linear patterns (i.e., U-shaped) have also been described[30], reflecting the flexibility of growth in response to changing environmental conditions[18]. Indeed, several events of growth suppression and release are usually experienced across the lifetime of tropical trees[31].

Most studies investigating forest biomass variations globally[32], regionally[33,34] and locally[35,36] use average wood specific gravity values at the species or the genus level extracted from global repositories such as DRYAD[7]. However, it has been shown locally that the use of such repositories[7,8] can lead to an overestimation of the wood specific gravity of approximately 16% for the species community[37]. Studies focused on biomass often neglect both “within genera” and “within species” variations in wood specific gravity, the latter potentially depending on tree size and mechanical constraints, or on environmental conditions. In contrast, studies describing the variability of wood specific gravity, and stressing the potential consequences of such variations on the biomass of the tree[26,30], barely consider biomass estimations in the framework of the carbon budget of the forest. Neglecting the variability of wood specific gravity might be essentially problematic for species that prominently contribute to forest biomass; i.e., species reaching large dimensions[32,38], or those that are frequent and/or locally ‘dominant’[33,39].

In the present study we aimed (i) to extract pith-to-bark wood specific gravity profiles of the 120 wood cores collected on 14 ‘dominant’ tree species, (ii) to characterize the variance partition of wood specific gravity profiles using linear mixed models and (iii) to quantify errors and biases in biomass estimations when wood specific gravity is not fully measured or only extracted from global repositories. To our knowledge, this is the first study addressing the problem of wood specific gravity radial variation in the framework of biomass estimations, i.e., with a particular attention on both intercepting detailed radial profiles and selecting species contributing mainly to the biomass of the forest.

## Material and Methods

### Data collection

The study area was located at the southern edge of the Congo Basin, north of the Bateke plateau, in the Bandundu province of the Democratic Republic of the Congo (WGS 1984; 2°29’35” S, 16°30’5.5” E). Mean annual temperature and mean annual rainfall are 25°C and 1500 mm.year^-1^, respectively, with a long dry season occurring between June and August and a short dry season in February[40]. The land cover is characterized as a forest-savanna mosaic[36,41] and forest species composition is identified as typical for Moist Central African forests[42]. Based on 26 1-ha plots sampled in 2011 and 2012 we estimated variations in above-ground biomass (AGB) between 27 and 460 Mg.ha^-1^ from young secondary to old-growth forests[36] (Table A in S2 File). The biomass of each tree with a diameter at breast height (dbh, measured at 130 cm or 50 cm above any buttresses) greater than or equal to 10 cm was estimated from the measurement of the dbh, total height and WSG using the pantropical equation developed for moist forests[2]. Measured trees were identified up to the species-level in the field, and samples were deposited in the Herbarium and botanical African library of the Université libre de Bruxelles (references Bastin-Serckx, #1–474). We selected 14 species from 14 genera and 8 families (Table 1) foremost based on their contribution to forest biomass but also considering contrasted regeneration guilds (7 non-pioneer light demanding, 5 shade-tolerant and 2 swamp species) as categorized by Hawthorne[43]. Non-pioneer light demanding species will be referred to as light-demanding species. The 14 species account for 50.7% of the total AGB estimated from inventory data[36] (Table B in S2 File). According to the global wood density repository registered on DRYAD [7,8] (registered under the following DOI: http://dx.doi.org/10.5061/dryad.234/1), average values for the 14 species range between 0.28 and 0.95 g.cm^-3^, with a mean of 0.65 g.cm^-3^.

**Table 1:** Characteristics of the 14 study species including botanical family, regeneration guild *sensu* Hawthorne[43], number of trees sampled (n), WSG, DBH, sample quantity (n) of the trees sampled, as well as frequency (% of plot presence), density (# of stems per hectare) and aboveground biomass (AGB) in the Malebo study site, in the Democratic Republic of Congo[36].

Species	Family	Guild	n	WSG g/cm³ (s.d.) [Min—Max]	DBH cm (s.d.) [Min—Max]	DBH max in plots	Freq in plots	Density (#stem/ha) in plots	AGB (t/ha) in plots	%AGB Tot in plots
*Dialium cf*.*pachyphyllum*	*Fabaceae (caesalpinoideae)*	SB	3	0.68(+/- 0.03)[0.66–0.71]	22.84 (+/- 14.47) [7.67–36.48]	125	21	3.50	9.80	3.64
*Entandrophragma angolense CJB 2011)*	*Meliaceae*	NPLD	6	0.59 (+/- 0.04) [0.53–0.62]	50.81 (+/- 16.12) [29.57–76.7]	126	56	6.00	3.21	1.19
*Gilbertiodendron dewevrei*	*Fabaceae (caesalpinoideae)*	SB	14	0.69 (+/- 0.04) [0.62–0.75]	33.01 (+/- 15.51) [12.39–65.87]	139	9	4.00	9.86	3.66
*Hallea stipulosa*	*Rubiaceae*	Swamp	7	0.44 (+/- 0.06) [0.31–0.47]	35.22(+/- 14.97)[15.52–50.78]	84	28	2.25	1.64	0.61
*Klainedoxa gabonensis*	*Irvigiaceae*	NPLD	3	0.90 (+/- 0.05) [0.85–0.95]	15.16 (+/- 6.34) [10.27–22.33]	160	84	8.09	43.10	16.00
*Macaranga staudtii*	*Euphorbiaceae*	Swamp / P	3	0.31 (+/- 0.03) [0.28–0.34]	23.36 (+/- 10.73) [12.46–33.92]	-	-	-	-	-
*Pentaclethra eetveldeana*	*Fabaceae (mimosoideae)*	NPLD	7	0.61 (+/- 0.04) [0.54–0.66]	41.51 (+/- 19.04) [13.78–68.14]	89	78	9.25	11.77	4.37
*Piptadeniastrum africanum*	*Fabaceae (caesalpinoideae)*	NPLD	14	0.70 (+/- 0.06) [0.60–0.80]	34.88 (+/- 12.72) [18.36–62.4]	175	41	0.84	5.57	2.07
*Plagiostyles africana*	*Euphorbiaceae*	NPLD	8	0.62 (+/- 0.04) [0.55–0.69]	36.26 (+/- 12.92) [17.33–50.15]	66	91	28.38	14.89	5.53
*Polyalthia suaveolens*	*Annonaceae*	SB	12	0.73 (+/- 0.05) [0.61–0.78]	30.65 (+/- 9.52) [15.17–47.04]	64	97	17.94	10.42	3.87
*Pycnanthus angolensis*	*Myristicaceae*	NPLD	9	0.52 (+/- 0.03) [0.49–0.56]	52.05 (+/- 23.42) [6.51–75.93]	150	56	5.81	5.69	2.11
*Staudtia kamerunensis*	*Myristicaceae*	SB	12	0.74 (+/- 0.04) [0.68–0.80]	42.81 (+/- 13.86) [26.75–69.96]	71	78	6.63	3.63	1.35
*Strombosia pustulata*	*Olacaceae*	SB	14	0.78 (+/- 0.08) [0.54–0.87]	33.46 (+/- 17.72) [11.16–77.12]	120	94	19.00	16.05	5.96
*Uapaca guineensis*	*Euphorbiaceae*	NPLD	8	0.71 (+/- 0.03) [0.66–0.77]	43.82 (+/- 12.53) [28.21–62.37]	85	6	9.84	0.84	0.31

**Fig 1:**
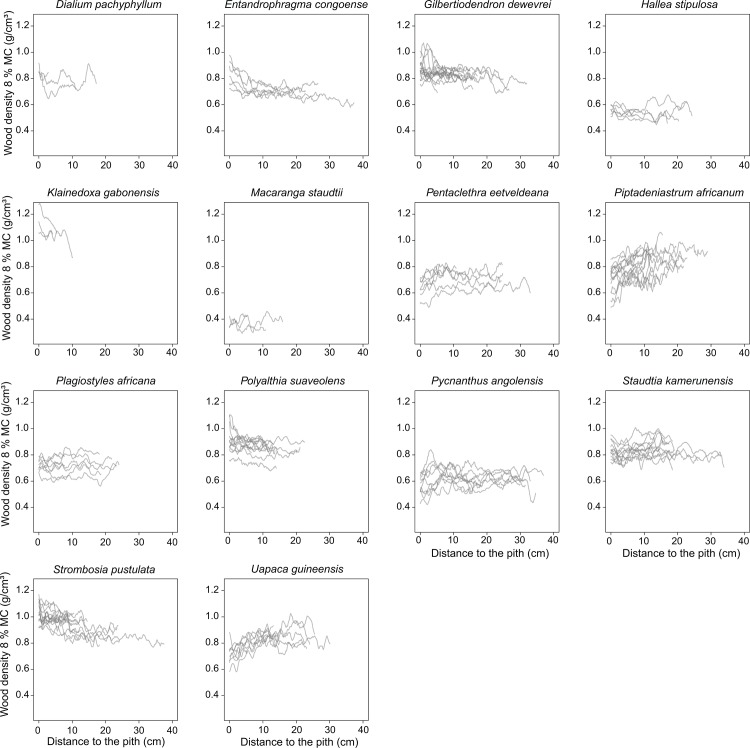
Variation in wood density measured at 8% of moisture content (g.cm^-^³) along the distance to the pith (cm) for the 14 species investigated in Malebo, the Democratic Republic of the Congo.

A total of 200 individual trees were sampled in the field. Because the pith was not noticeable on 80 of the 200 cores scanned (S1 File), these were excluded from further analyses. Remaining samples covered a wide range of diameters (from 6.5 to 77 cm, i.e., pith-to-bark profile from 3.25 to 38.5 cm), with 3 to 14 sampled trees per species (Table 1). Wood cores were extracted manually using Häglof Pressler augers of 30 or 60 cm length and 5 mm diameter. No specific permissions were required for the extraction of wood cores because these constitute only superficial and non-destructive vegetative samples from non-endangered species. We measured the wood density at 8% moisture content (WD_8%_) along the radial profile of each wood core, i.e., from pith-to-bark, using an X-ray CT scanner built at the Ghent University Centre for X-ray Tomography (UGCT; http://www.ugct.ugent.be). Cores were scanned using a closed microfocus X-ray tube to obtain a profile resolution of 50 μm[44] and extracted as wood density profiles using the Fiji software 1.6.0_24. Core snapshots reconstructed with a cross section view allowed for the identification of the exact location of the pith (Figs A and B in S2 File). For each core, a 1-D microdensitometric profile from pith-to-bark was extracted and smoothed at 1-mm resolution using a moving average window (Fig 1).

### Data analysis

We first used the linear mixed framework to identify variations in the radial profile[45]. Regeneration guild and distance to the pith were included as fixed effects whereas species and individual within species were treated as random effects (see Eq 1). This modeling approach accounts for dependencies between measurements on the same species and individuals. As variance components for random slopes cannot be easily integrated with other variance components[46], we only considered random intercepts.

$$WD_{lkji}\;=\;\beta_{0l}+\;\beta_{1l}d_{ij}+\alpha_{0ij}+\alpha_{1ijk}\;+\;\varepsilon_{ijkl}$$ (equation 1)

where *WD*_*lkij*_ is the wood density (g.cm^-^³) for the *i*th 1-mm interval in the *j*th core of the *k*th species and the *l*th regeneration guild; *d*_*ij*_ is the radial distance from the i*th* interval to the pith of the j*th* core (in mm); *β*_0_ and *β*_1_ are the fixed effects; *α*_0_ and *α*_1_ are the random intercepts; and *ε*_*ijkl*_ is the residual variance within individual. Model diagnostics, i.e., variance constancy, homogeneity and normality, were assessed graphically[47]. Model selection was undertaken using likelihood ratio tests against several reduced models (see Table 2 [48]), completed by Akaike criterion and Kenward-Roger’s analysis of variance for fixed effects (Table C in S2 File).

**Table 2:** Coefficients from Gaussian linear mixed models predicting the wood density along the radial profile for the 14 species investigated in Malebo, the Democratic Republic of Congo. Coefficient estimates are provided for the fixed effects (at 95% of confidence interval). The contribution to the total variance is estimated for the random effect through the maximum of likelihood.

Model name	Null model (without fixed effect)	Reduced Model	Full Model
Fixed effects			
intercept	0.764	0.766	0.761
Shade-tolerant	-	0.112	0.116
Swamp	-	-0.317	-0.307
Distance to the pith	-	0.001	0.002
Shade-tolerant * Distance to the pith	-	-0.004	-0.004
Swamp * Distance to the pith	-	0	-0.001
% of variance	0%	44%	36%
Random effects	Estimated contribution to the total variance	Estimated contribution to the total variance	Estimated contribution to the total variance
Species	84%	45%	45%
Species|individuals	8%	-	11%
Residuals	8%	11%	8%
AIC	-64297	-52378	-65835
Log likelihood ratio-test vs. Full Model	P < 0.001	P < 0.001	-

Then, we calculated the mean WSG of each tree accounting for the increasing surface of wood along the profile[49], hereafter referenced as the weighted-WSG. We used the conversion relationship developed by Sallenave[50] to convert WD_8%_ in WSG:

$$WSG\;=\frac{D-Md}{1+v\;\left(\;S-M\;\right)}$$ (equation 2)

where *D* is the wood density measured at moisture content *M*; *d* is the correction of *D* when the moisture content varies by 1%; *v* is the variation of the wood volume when the moisture content varies by 1%; and *S* is the fiber saturation point. Average *d*, *v* and *S* for each species were obtained from literature data on wood physical and mechanical properties[50–52]. Species not recorded were converted to WSG using the coefficients from the relationship between WSG and WD_8%_ fitted on recorded species. This relationship is very stable and is comparable to previous work performed at a larger scale[14] with a slope of 0.860 and an intercept close to 0 (n = 94; r² = 0.987; P<0.001).

We tested the validity of the WSG derived from the scans by comparing the results with WSG measured from the water displacement method[13] for a random subset of 25 wood cores belonging to 10 of the 14 study species (Figure C in S2 File). Samples were oven-dried at 105°C for 48 to 72 h. Dry mass and dry volume displacement were measured on a 1 mg precision scale and converted in WSG using Eq 2. We found a systematic bias (~ 9%; R² = 0.99; Figure C in S2 File), and consequently applied a systematic correction on the 120 wood cores[44]. This correction has been performed using the parameters of the linear relationship between WSG measured from 3D X-rays and WSG measured by water displacement method (Figure C in S2 File).

We used paired t-test to determine any significant differences between the mean WSG of the 14 species between weighted and the global datasets, and we used linear regression analysis to identify potential biases.

The potential error in WSG and AGB resulting from ignoring WSG radial variations was assessed by comparing the weighted-WSG, used as the reference, with the WSG from partial samples[26], i.e., the inner-WSG (2 cm from the pith) and the outer-WSG (2 cm under the bark). We then calculated the slope between the inner-WSG and the outer-WSG and estimated the influence on the potential error in WSG at the individual, species, and regeneration guild level. These slopes indicate the importance of radial variations[53]. Slopes were scaled-up to meters (multiplied by 100) to simplify interpretation, such that they express the variation of WSG in g.cm^-3^ for a radial length of 1 meter[26].

All statistical analyses were performed within the R environment (R Development Team) using the lmer() function of the lme4 and lmerTest packages[54].

## Results

### Pith-to-bark profiles

We first examined the major variations in WSG. On Fig 1, we can see positive trends on 3 of the light-demanding species (*Piptadeniastrum africanum*, *Uapaca guineensis* and *Pentaclethra eetveldeana*) and negative trends on 2 light-demanding species (*Klainedoxa gabonensis*, *Entandrophragma angolense*), and 3 shade-bearer (*Gilbertiodendron dewevrei*, *Polyalthia suaveolens* and *Strombosia pustulata*). However, as only 3 samples were recorded on *K*.*gabonensis*, such trends need to be interpreted with caution. The rest of the species examined presented no obvious global trends.

### Linear mixed models and variance partition

**Fig 2:**
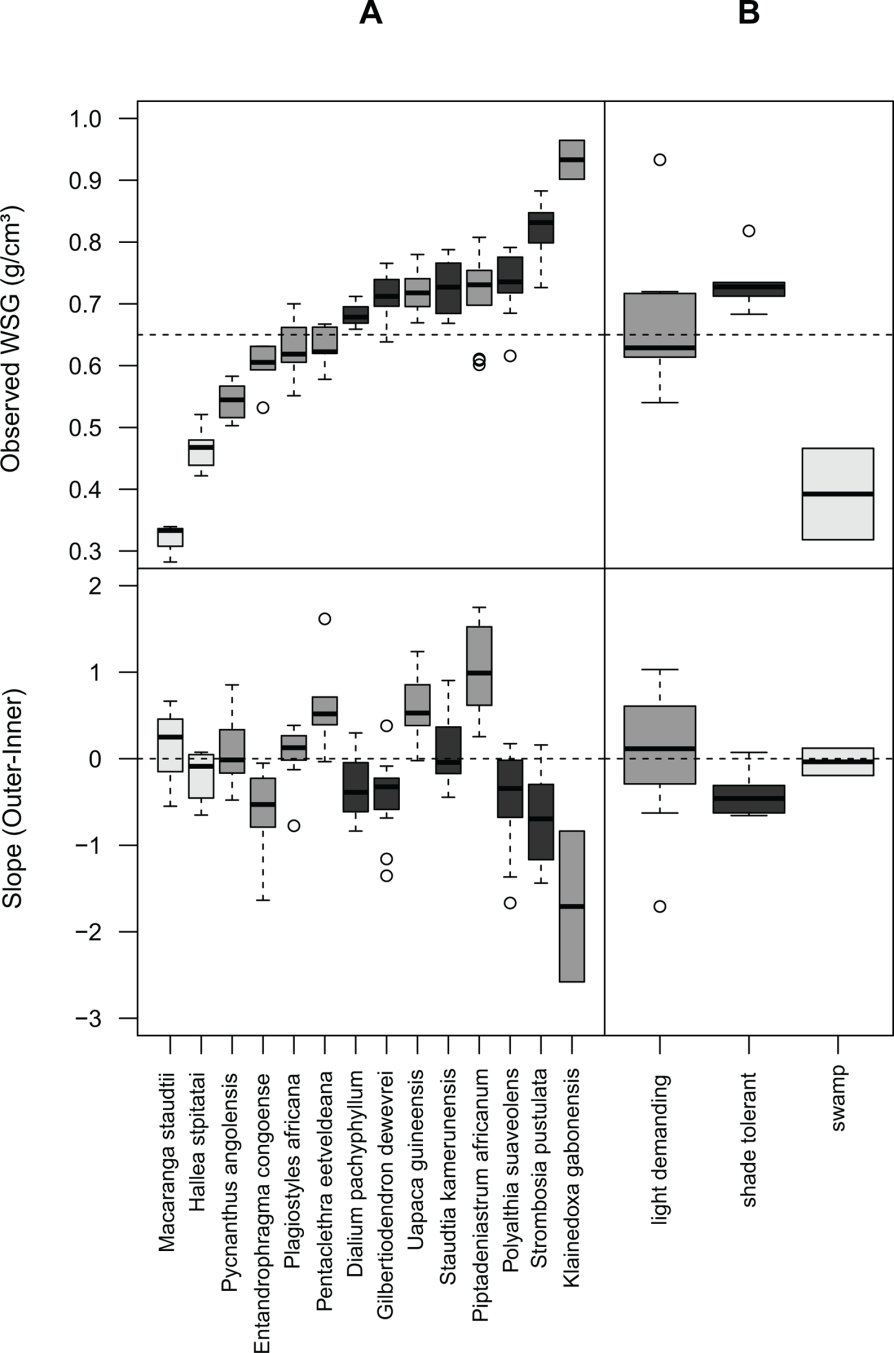
Boxplots of the WSG and of the slope calculated between the outer-WSG and the inner-WSG for the 14 species (A) and the corresponding regeneration guilds (B) investigated in Malebo, the Democratic Republic of the Congo. The color corresponds to the regeneration guild with swamp, light-demanding and shade-tolerant species respectively colored in light grey, grey and dark grey, respectively.

The best model, regarding AIC and LRT, accounted for regeneration guilds and nested effect between species and individuals (Table 2). This model explained 92% of the total variability in wood density. Species alone explained 45%, with an additional 36% explained both by species and regeneration guilds. Individuals alone explained 11%. The studied species showed contrasting mean WSG values, ranging from 0.35 for the light-wooded *Macaranga staudtii* to 0.93 g.cm^-^³ for the dense-wooded *Klainedoxa gabonensis* (Fig 2A). When aggregating species by regeneration guild (Fig 2B), we found that swamp species had the lowest mean WSG (~0.35 g.cm^-^³) while shade-tolerant species had WSG significantly above the average (~0.71 g.cm^-^³). Light-demanding species did not show any particular pattern (~0.63 g.cm^-^³) with values oscillating around the average of the 14 species (~0.65 g.cm^-^³).

We then analyzed radial variations in WSG based on the comparison of the weighted-WSG along the radial profile and the WSG from DRYAD. We showed that average weighted-WSG for the 14 species of the 120 trees investigated was not significantly different from the average WSG calculated from DRYAD (t = -0.1539; df = 24.635; P = 0.88; Figure D in S2 File). However, we observed a substantial bias (intercept = 0.17; slope = 0.74), with the underestimation of WSG for light-wooded species and an overestimation for dense-wooded species when using WSG from DRYAD.

### Errors and biases in biomass estimations

**Fig 3:**
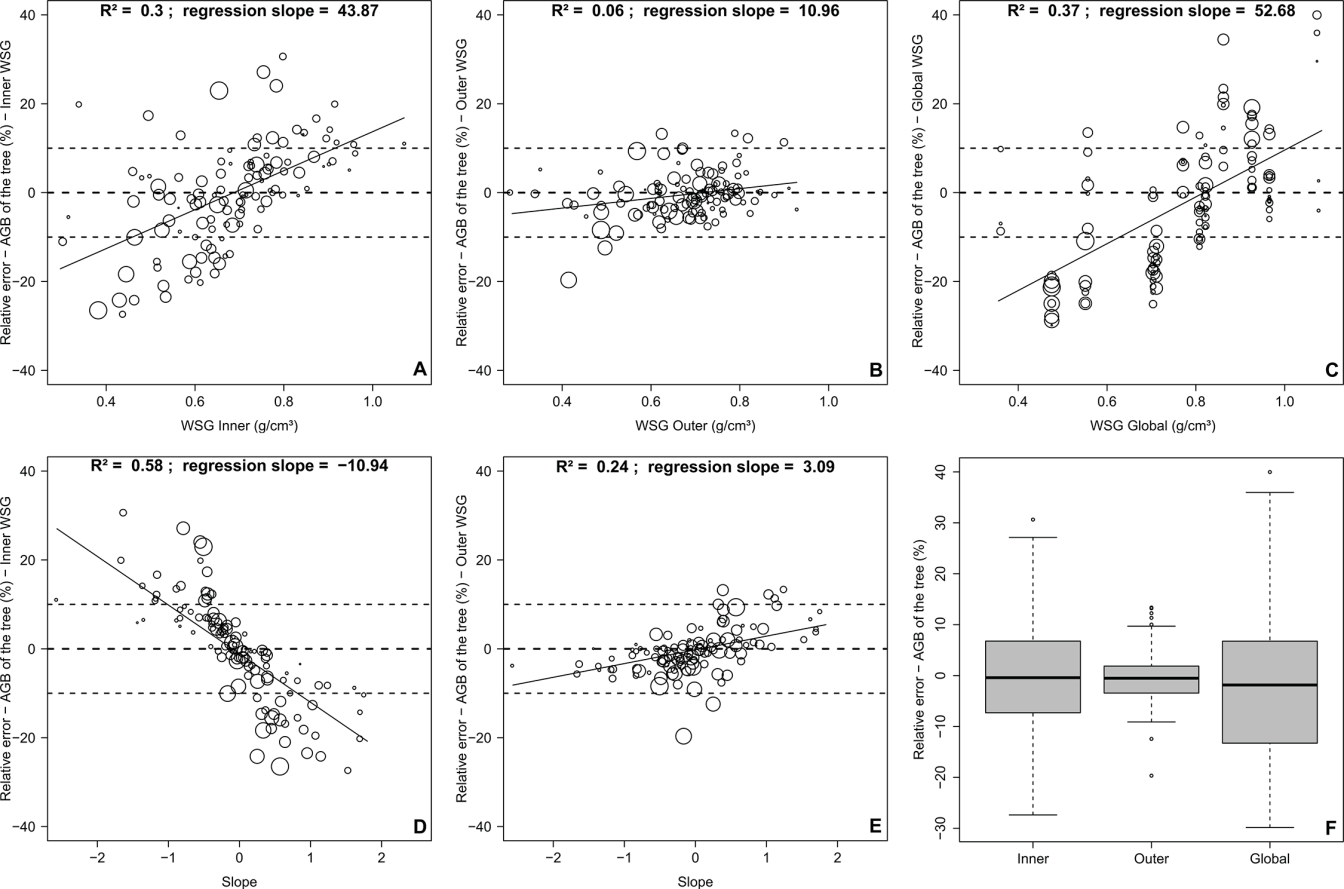
Relative error of the estimation of the AGB for each sampled tree in Malebo, the Democratic Republic of the Congo. Relative errors were calculated using weighted-WSG as reference and using the inner-WSG (A,D), the outer-WSG (B,E) and the global-WSG (C) as estimators. The dependence of the relative error was tested against the absolute value of WSG and against the slope. The size of the dots is proportional to tree diameter. The final boxplots summarize the distribution of the errors according to each estimator (F).

Finally, we quantified the errors in AGB estimations when inner-WSG, outer-WSG or WSG from DRYAD are used as a proxy of WSG (Fig 3), using as reference the AGB estimated with the weighted-WSG. In addition, we analyzed the relationship between the error in AGB, the WSG (from inner, outer and DRYAD) and the slope between the inner and the outer WSG for each single tree sampled. Both the use of inner-WSG and WSG from DRYAD revealed substantial errors (Fig 3A, 3C and 3D). The use of the inner-WSG presented an error strongly related to the slope (Fig 3D), with an underestimation of WSG for light-wooded species and an overestimation for dense-wooded species (Fig 3A). Interestingly, using the outer-WSG yielded much less error with no significant dependence on tree WSG or slope (Fig 3B and 3E). In the end, regarding all the 120 trees together, we found the error consequently averages out, whatever the proxy of WSG used (Fig 3F).

**Fig 4:**
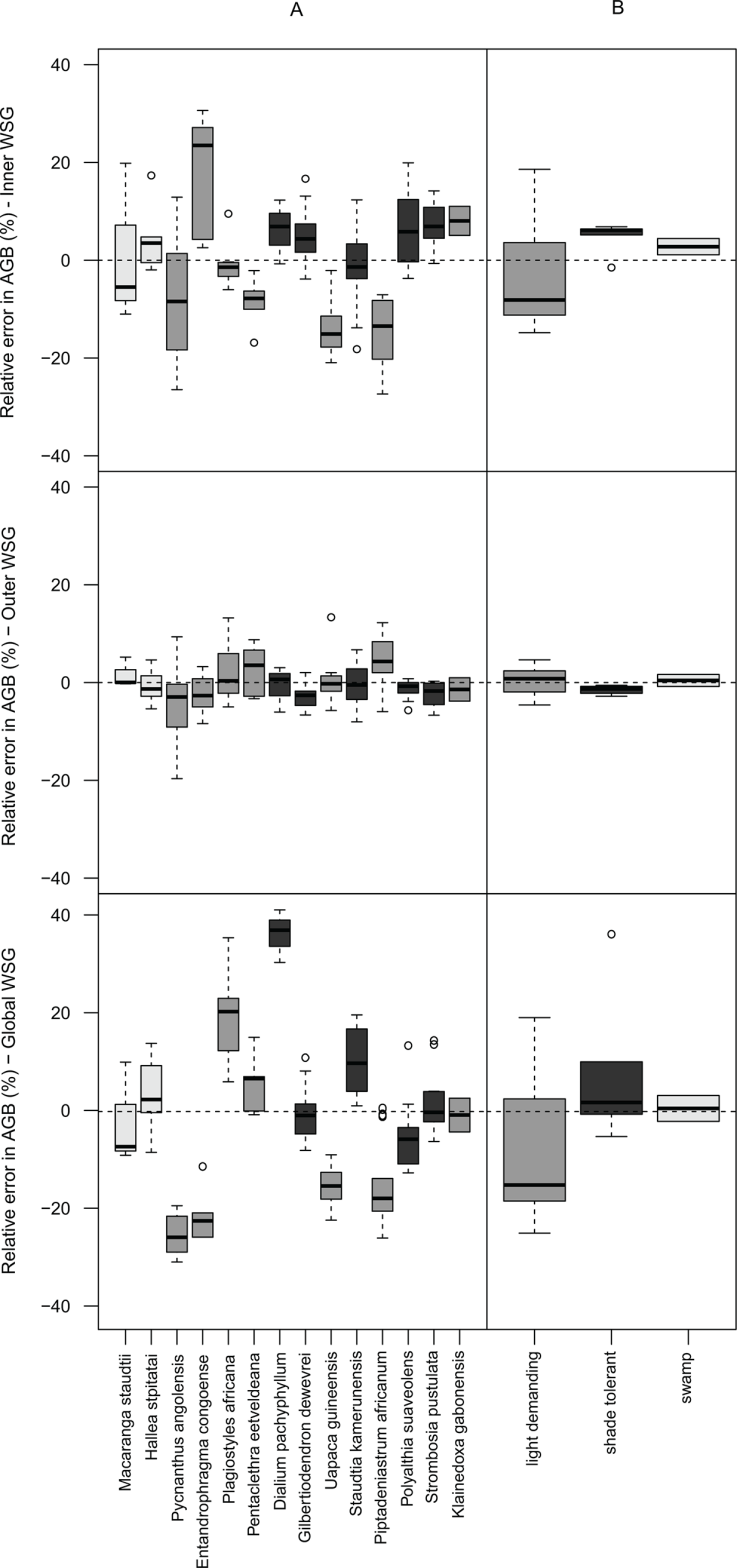
Boxplots of the relative errors calculated for the 14 species (A) and the three regeneration guilds (B) sampled in Malebo, the Democratic Republic of the Congo. Relative errors were calculated using weighted-WSG as reference and using the inner-WSG, the outer-WSG and the global-WSG as proxies. Swamp, light-demanding and shade-tolerant species are respectively colored in light grey, grey and dark grey, respectively.

Regarding species and guilds (Fig 4), we found that the AGB estimated from inner-WSG tended to be overestimated for shade-tolerant species while no general trend was identified for light-demanding species. In particular, the WSG and AGB of *E*. *angolense* and *K*. *gabonensis* tended to be overestimated while *P*. *africanum* and *U*. *guineensis* tended to be underestimated. Interestingly, no specific or general pattern was observed when using the outer-WSG. When using global-WSG, no general trends were found but the error at species level was considerable. Specifically, the use of global-WSG values for *Dialium pachyphyllum* led to 40% overestimation of the AGB, followed by *Pycnanthus angolensis* (-26%), *E*. *angolense* (-23%), and *Plagiostyles africana* (+20%).

## Discussion

The first aim of our study was to examine the variation in WSG among and within Central African tree species, including radial variation within individuals. Based on an extensive analysis conducted at local scale, we found that species explained most wood density radial variance whereas individuals explained only a minor part. These results are consistent with previous observations in Panama[30] and Australia[55]. In addition, we found that the share of variance generally explained by species only (>70%) can actually be partitioned into 45% explained only by species, 36% shared with regeneration guilds, and 11% explained only by individuals. This observation highlights the strong relationship between wood density and regeneration guild and supports the idea of an integrated wood economic spectrum [8,17].

Shade-tolerant species systematically showed high WSG values with a decreasing trend along the radial profile (negative slopes, Fig 4). This observation supports assumptions about slow growth and investment in dense wood by shade-tolerant species, in particular during the early stages of their life[21,22]. The decrease of wood density from pith-to-bark is often attributed to increased light exposure and improved growth conditions in the later stages[19] with possible access to the canopy for some trees during ontogeny[31].

Wood density profiles with an increasing trend from pith-to-bark and a low WSG were not systematic for light-demanding species. Only three species out of seven showed an increasing profile: *P*.*africanum*, *U*.*guineensis* and *Pentaclethra eetveldeana*. In the study area, *U*.*guineensis* is the dominant species in young secondary forests colonizing the savannas[36] and *P*.*eetveldeana* and *P*.*africanum* both exhibit a distribution of diameters with a low relative abundance of small trees indicative of a regeneration shortage (Figure E in S2 File). These three species are unlikely to persist in old-growth forests, supporting the assumption that strictly light-demanding species present increasing trends of WSG from pith-to-bark[18,20]. The other light-demanding species showed a reverse-J shaped relationship between tree size and frequency (Figure E in S2 File), characteristic of species well established in a mature forest and tolerant to shade[56]. This confirms that the regeneration guild of a species may vary between sites[43] and should therefore be attributed with caution.

The second aim of our study was to assess the potential error in AGB estimations resulting from radial variations in WSG and from intraspecific variations due to differences between observed and global-WSG values. Most importantly, we found radial variations have minor consequences for AGB estimations as the outer-WSG is strongly correlated to the weighted-WSG at the individual (RMSE of 3.7% and error centered on 0), the species, and the regeneration guild level. This result is mainly explained by the 3-D geometrical properties of trees: the outer wood occupying more volume than the inner wood in a tree and being consequently much more representative of the weighted-WSG. For instance, for a tree with a dbh of 30 cm and assuming a perfect circular shape, the volume of outer wood (2 cm under the bark; 91 cm²) is 30-fold larger than the volume of inner wood (2 cm from the pith; 3 cm²). To evaluate the potential generalization of this result, we compared outer-WSG with weighted-WSG of published datasets from Costa Rica[26]. We found a strong correlation between the two (intercept = -0.03; slope = 1.05; Figure F in S2 File), which reinforce that the outer-WSG can safely be used as an estimator of the weighted-WSG, regardless of the location. Consequently, intersecting the full radial profile of WSG is not required to properly estimate the AGB of a tree. However, as tree growth is a dynamic process[57], further consideration should be given to the variations of the carbon stored within a forest through time. In that context, radial profiles of WSG acquired at a very high spatial resolution constitute a great opportunity to improve our understanding of the relationship between tree growth, tree dimension and tree anatomical properties[58].

We also found that the use of WSG from global repositories can strongly affect the estimation of AGB depending on the species concerned (up to 40% for *Dialium pachyphyllum*). Because radial variations do not explain such bias in AGB estimations, we suggest that this may result from intraspecific variation for widely distributed species occurring under various climatic regimes. WSG from global repositories must consequently be used with caution when estimating the AGB of these species. Consequently, to minimize the potential error in AGB estimations related to the WSG, we recommend the extraction of a superficial sample of wood (2 cm under the bark). Considering that a small proportion of tropical tree species disproportionately contributes to the regional stem abundance[59] and biomass[33], we particularly recommend the systematic collection of outer wood samples for these ‘biomass dominant’ species. This should offer new opportunities to study the intraspecific variation of WSG along bioclimatic gradients.

## Supporting Information

S1 File Dataset of the detailed pith-to-bark profiles. (TXT)

S2 File Supporting informations. Detailed wood density profile illustration with X-ray **(Figure A)**, Pith localization **(Figure B)**, Xray and water displacement correlation **(Figure C)**, Dryad vs. observed values **(Figure D)**, Species local diametric structures **(Figure E)**, Outer WSG vs. inner WSG wood as proxy of weighted WSG in Costa Rica **(Figure F)**, Biomass inventories metadata **(Table A)**, Species contribution to total biomass **(Table B)**, Kenward-Rogers approximation **(Table C)**.
(DOC)
