# Supplementary material for: Wood Specific Gravity Variations and Biomass of Central African Tree Species: The Simple Choice of the Outer Wood
Source: PLoS One. 2015 Nov 10;10(11):e0142146. doi: 10.1371/journal.pone.0142146 (PMC4640573; doi:10.1371/journal.pone.0142146)
Supplement: S2 File — Detailed wood density profile illustration with X-ray (Figure A), Pith localization (Figure B), Xray and water displacement correlation (Figure C), Dryad vs. observed values (Figure D), Species local diametric structures (Figure E), Outer WSG vs. inner WSG wood as proxy of weighted WSG in Costa Rica (Figure F), Biomass inventories metadata (Table A), Species contribution to total biomass (Table B), Kenward-Rogers approximation (Table C). (DOC) [file pone.0142146.s002.doc]

**S2 File.**

**
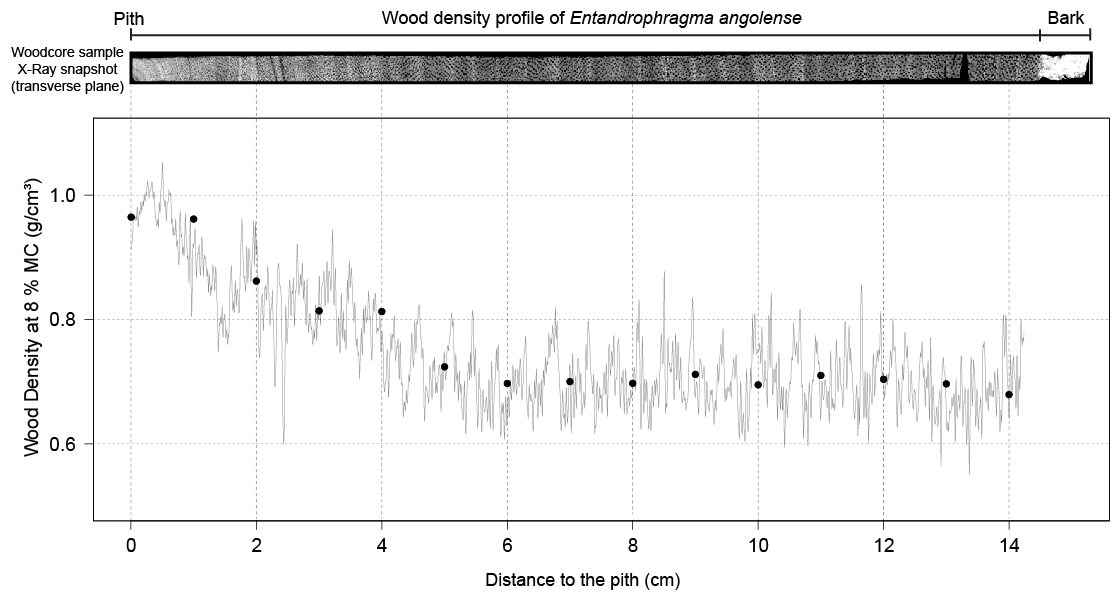
**

**S2 File. Figure A.** **Detailed wood density profile using X-ray**. Illustration of the 1-D microdensitometric profile of the wood density measured at 8 % of moisture content (g.cm^-^³) for a core of *Entandrophragma angolense*. The profile shows a decreasing trend near the pith which is stabilized after 5 cm. Black-dotted points represent the mean wood density value along the profile extracted with a 1 cm moving window.

**
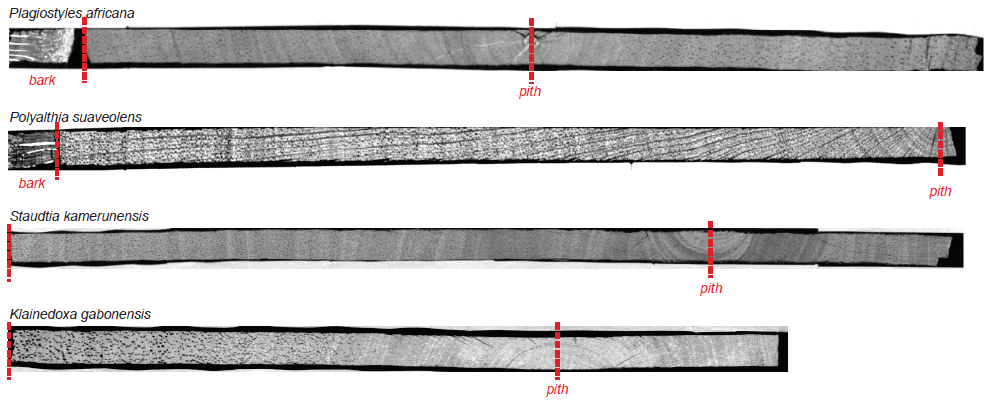
**

Supplementary Figure 2

**S2 File. Figure B**. **Pith localization**. Illustration of the localization of the pith based on the visual interpretation of 3D X-ray scans performed on woodcores extracted from *Plagiostyles Africana*, *Polyalthia suaveolens*, *Staudtia kamerunensis*, *Klainedoxa gabonensis*. The shades of grey, from dark to light grey, represent the range of wood density, from low to high values.

**
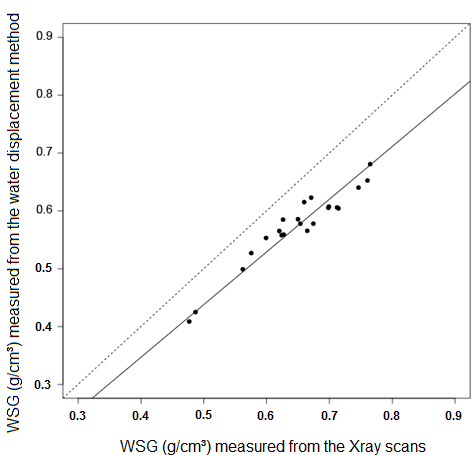
**

**S2 File. Figure C.** **Xray and water displacement correlation**. Correlation between the weighted-WSG measured from 3-D Xray scans with the WSG measured by the water displacement method for a subset of 25 wood cores sampled randomly and accounting for 10 of the 14 species investigated in Malebo, the Democratic Republic of the Congo.. The dotted line represents the perfect correspondence (1:1) and the full line is the linear regression between WSG measured from Xray. A systematic overestimation is observed with X-ray analysis (slope= 0.911, R²=0.92).

**
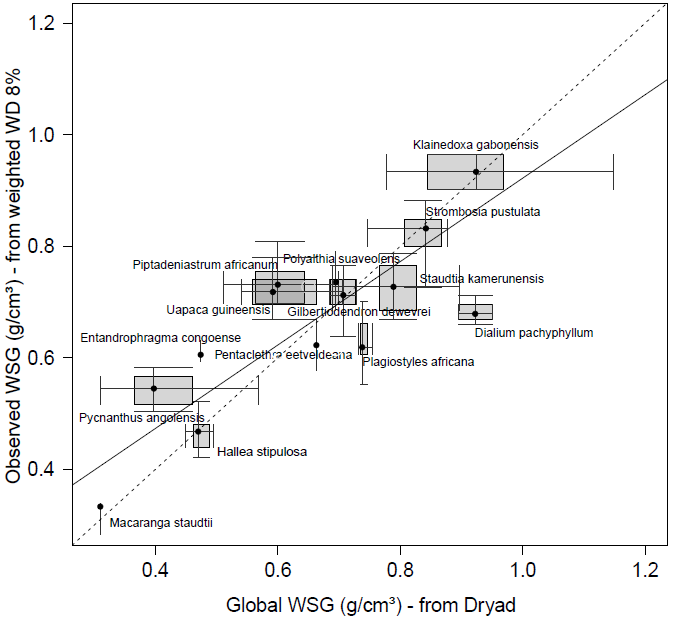
**

**S2 File. Figure D. Dryad vs. observed values.** Double boxplots presenting the correspondence between the weighted-WSG measured from 3-D Xray scans with WSG extracted from the Dryad repository (Zanne 2009) for the 14 species investigated in Malebo, the Democratic Republic of the Congo. The dotted line represents the perfect correspondence (1:1) and the full line is the linear regression between global-WSG and weighted-WSG (intercept=0.17; slope = 0.74; R²=0.83).

**
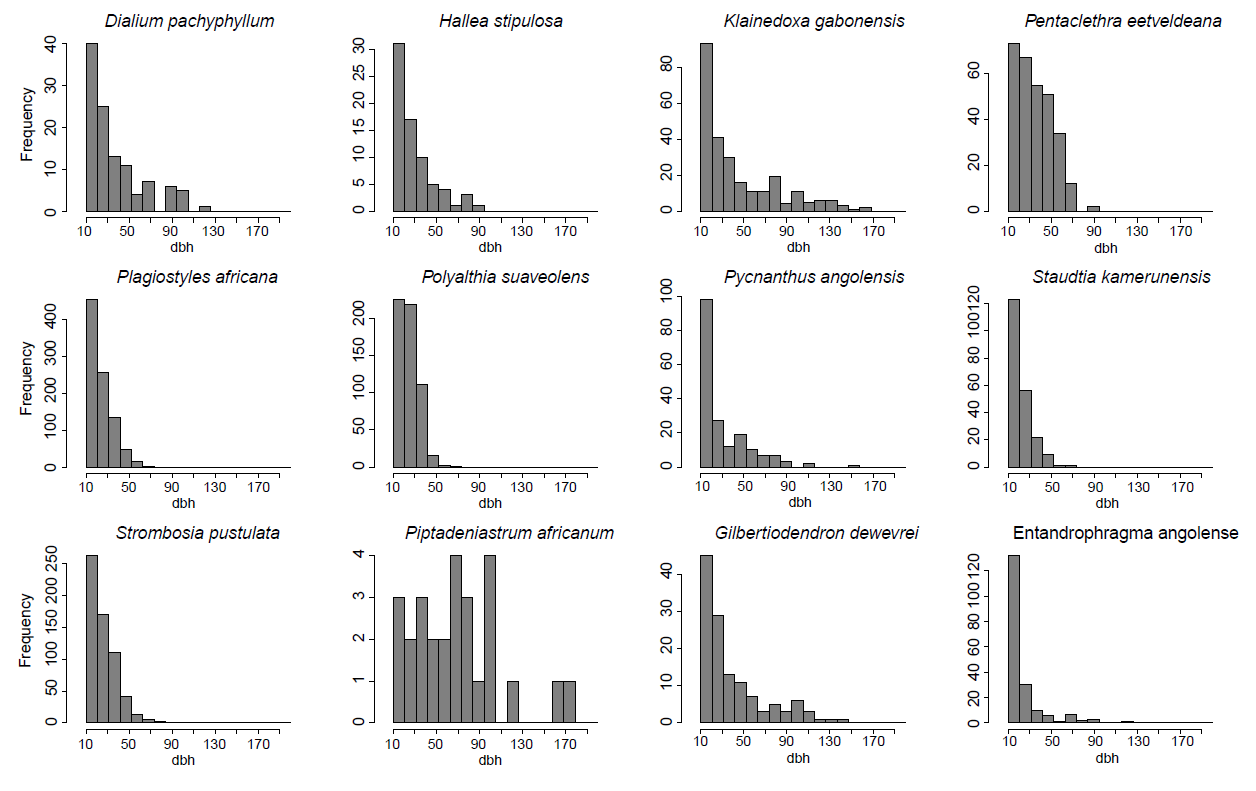
**

**S2 File. Figure E.** **Species local** **diametric structures.** Distribution of diameters of the 14 species studies in the 26 1-ha plots sampled in the Malebo site.

**
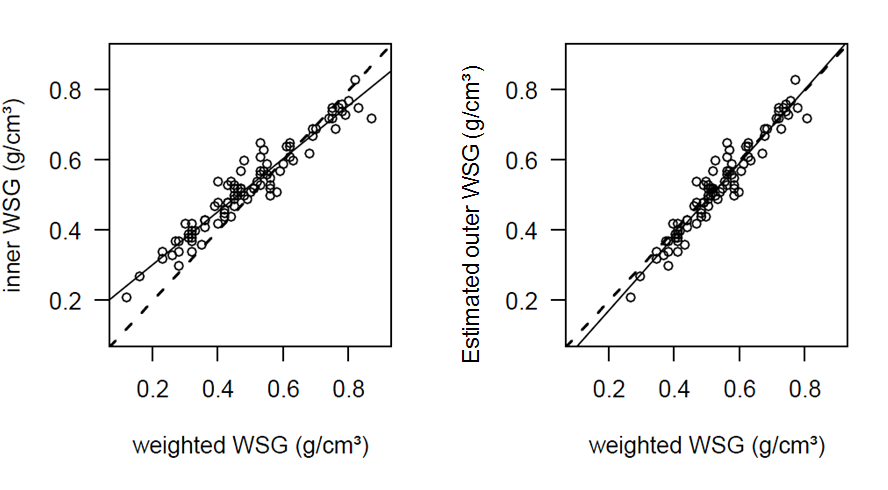
**

**S2 File. Figure F. Outer WSG vs. inner WSG wood as proxy of weighted WSG in Costa Rica.** Relationship between the inner-WSG and the estimated outer-WSG with the weighted-WSG for the XX species investigated by Plourde et al. (2015) in Costa Rica. The results show the bias is very low when using the outer-WSG (intercept = -0.03; slope = 1.05) compared to the inner-WSG (intercept = 0.15 ; slope = 0.76) as a proxy of the weighted-WSG.

The values of weighted-WSG and inner-WSG were extracted from their published supplementary material. As they calculated the radial slope based on the relationship between the inner-WSG and the outer-WSG we assumed the inner-WSG was equal to their intercept. Outer-WSG was then estimated based on the relationship between the inner-WSG and the outer-WSG developed in their study.

**S2 File. Table A. Biomass inventories metadata.** Forest structural properties (biomass, basal area, maximum DBH, mean DBH, stem density) of the 26 1-ha plots inventoried in 2011 and 2012 in the northern of the Bandundu province, the Democratic Republic of the Congo.

| plot-ref | Lat | Long | stem density | mean DBH | maximum DBH | Basal Area | Aboveground biomass |
| --- | --- | --- | --- | --- | --- | --- | --- |
| M1P1 | -2 31 41.623 | 16 28 24.949 | 386 | 24.69 | 96.2 | 26.40 | 345.36 |
| M1P2 | -2 32 46.740 | 16 28 25.573 | 181 | 33.36 | 140 | 22.16 | 311.98 |
| M1P3 | -2 32 32.950 | 16 30 5.201 | 279 | 24.93 | 175 | 25.40 | 327.25 |
| M1P4 | -2 33 16.908 | 16 30 2.144 | 349 | 27.08 | 150 | 31.07 | 400.23 |
| M1P5 | -2 34 7.795 | 16 28 40.298 | 350 | 24.67 | 175 | 26.31 | 287.10 |
| M1P6 | -2 35 45.796 | 16 28 40.606 | 477 | 24.32 | 143 | 33.83 | 357.12 |
| M1P7 | -2 36 7.906 | 16 29 6.466 | 473 | 25.56 | 99.5 | 34.71 | 337.54 |
| M1P8 | -2 36 19.370 | 16 28 8.238 | 385 | 23.91 | 160 | 29.10 | 395.95 |
| M1P9 | -2 36 51.238 | 16 28 42.236 | 318 | 25.34 | 206 | 28.82 | 412.39 |
| M1P10 | -2 36 51.519 | 16 29 19.047 | 458 | 23.82 | 138 | 33.18 | 409.92 |
| M1P11 | -2 37 55.953 | 16 29 45.768 | 378 | 24.46 | 150 | 29.65 | 461.42 |
| M1P12 | -2 35 11.799 | 16 25 56.556 | 313 | 24.64 | 126 | 21.09 | 243.06 |
| M2P1 | -2 24 59.686 | 16 30 49.457 | 447 | 23.40 | 88 | 24.47 | 285.89 |
| M2P2 | -2 24 57.722 | 16 30 58.939 | 476 | 23.35 | 120 | 26.76 | 299.85 |
| M2P3 | -2 25 57.875 | 16 32 9.411 | 462 | 18.80 | 120 | 17.35 | 161.84 |
| M2P4 | -2 26 3.739 | 16 32 6.213 | 305 | 21.51 | 120 | 16.75 | 183.12 |
| M2P5 | -2 27 20.850 | 16 32 50.518 | 319 | 25.07 | 124 | 25.47 | 284.48 |
| M2P6 | -2 27 19.214 | 16 32 57.249 | 267 | 25.90 | 96 | 18.70 | 189.98 |
| M2P7 | -2 27 28.273 | 16 33 46.623 | 351 | 20.27 | 59.2 | 13.78 | 112.57 |
| M2P8 | -2 27 24.032 | 16 33 53.675 | 336 | 24.81 | 168 | 27.27 | 331.15 |
| M2P9 | -2 23 42.906 | 16 34 45.728 | 139 | 36.06 | 96.2 | 19.12 | 198.56 |
| M2P13 | -2 23 43.536 | 16 35 4.178 | 380 | 23.95 | 84 | 21.00 | 175.53 |
| M2P12 | -2 23 41.566 | 16 35 17.900 | 110 | 39.78 | 111 | 19.09 | 220.39 |
| M2P11 | -2 23 31.148 | 16 35 17.920 | 426 | 22.06 | 89.6 | 21.55 | 188.47 |
| M2P10 | -2 23 51.356 | 16 34 58.458 | 356 | 25.01 | 107.9 | 24.03 | 235.14 |
| M2P14 | -2 28 35.866 | 16 30 56.142 | 378 | 15.76 | 43.2 | 8.19 | 27.45 |
| M2P15 | -2 28 39.112 | 16 31 4.173 | 415 | 18.94 | 62 | 14.57 | 124.96 |

**S2 File. Table B**. **Species contribution to total biomass**. Species ranking according to their contribution to forest biomass in the 26 1-ha plots inventoried in 2011 and 2012 in the northern of the Bandundu province, the Democratic Republic of the Congo.

| Rank | *Species* | *Family* | AGB | AGB perc | AGB perc cum |
| --- | --- | --- | --- | --- | --- |
| 1 | ***Klainedoxa gabonensis*** | *Irvingiaceae* | 43.10 | 16.17 | 16.17 |
| 2 | *Millettia laurentii* | *Fabaceae* | 16.27 | 6.10 | 22.27 |
| 3 | ***Strombosia pustulata*** | *Olacaceae* | 16.05 | 6.02 | 28.29 |
| 4 | ***Plagiostyles africana*** | *Euphorbiaceae* | 14.89 | 5.58 | 33.87 |
| 5 | ***Pentaclethra eetveldeana*** | *Fabaceae* | 11.77 | 4.42 | 38.29 |
| 6 | ***Polyalthia suaveolens*** | *Annonaceae* | 10.42 | 3.91 | 42.20 |
| 7 | ***Gilbertiodendron dewevrei*** | *Fabaceae* | 9.86 | 3.70 | 45.90 |
| 8 | ***Dialium cf. pachyphyllum*** | *Fabaceae* | 9.80 | 3.68 | 49.57 |
| 9 | *Scorodophloeus zenkeri* | *Fabaceae* | 5.87 | 2.20 | 51.77 |
| 10 | ***Pycnanthus angolensis*** | *Myristicaceae* | 5.69 | 2.14 | 53.91 |
| 11 | ***Piptadeniastrum africanum*** | *Fabaceae* | 5.57 | 2.09 | 56.00 |
| 12 | *Drypetes spp.* | *Putranjivaceae* | 5.42 | 2.03 | 58.03 |
| 13 | *Copaifera spp.* | *Fabaceae* | 5.33 | 2.00 | 60.03 |
| 14 | *Ongokea gore* | *Olacaceae* | 5.26 | 1.97 | 62.00 |
| 15 | *Bikinia evrardii* | *Fabaceae* | 4.86 | 1.82 | 63.83 |
| 16 | *Pentaclethra macrophylla* | *Fabaceae* | 4.84 | 1.82 | 65.64 |
| 17 | *Strombosiopsis tetrandra* | *Olacaceae* | 4.71 | 1.77 | 67.41 |
| 18 | *Brachystegia laurentii* | *Fabaceae* | 4.07 | 1.53 | 68.94 |
| 19 | ***Staudtia kamerunensis*** | *Myristicaceae* | 3.63 | 1.36 | 70.30 |
| 20 | ***Uapaca guineensis*** | *Phyllanthaceae* | 4.34 | 1.63 | 71.92 |
| 21 | *Symphonia globulifera* | *Clusiaceae* | 3.30 | 1.24 | 73.16 |
| 22 | *Garcinia punctata* | *Clusiaceae* | 3.29 | 1.24 | 74.40 |
| 23 | ***Entandrophragma angolense*** | *Meliaceae* | 3.21 | 1.21 | 75.60 |
| 24 | *Santiria trimera* | *Burseraceae* | 3.03 | 1.14 | 76.74 |
| 25 | *Sorindeia africana* | *Anacardiaceae* | 2.76 | 1.04 | 77.78 |
| 26 | *Xylopia aethiopica* | *Annonaceae* | 2.75 | 1.03 | 78.81 |
| 27 | *Milicia excelsa* | *Moraceae* | 2.50 | 0.94 | 79.74 |
| 28 | *Duvigneaudia inopinata* | *Euphorbiaceae* | 2.43 | 0.91 | 80.66 |
| 29 | *Chaetocarpus africanus* | *Euphorbiaceae* | 2.34 | 0.88 | 81.54 |
| 30 | *Heisteria parvifolia* | *Olacaceae* | 2.32 | 0.87 | 82.41 |
| 31 | *Baillonella toxisperma* | *Sapotaceae* | 2.14 | 0.80 | 83.21 |
| 32 | *Chrysophyllum lacourtianum* | *Sapotaceae* | 2.09 | 0.78 | 83.99 |
| 33 | *Entandrophragma utile* | *Meliaceae* | 1.90 | 0.71 | 84.70 |
| 34 | *Trichilia rubescens* | *Meliaceae* | 1.90 | 0.71 | 85.42 |
| 35 | *Rytigynia spp.* | *Rubiaceae* | 1.89 | 0.71 | 86.13 |
| 36 | *Dacryodes edulis* | *Burseraceae* | 1.72 | 0.65 | 86.77 |
| 37 | ***Hallea stipulosa*** | *Rubiaceae* | 1.64 | 0.61 | 87.39 |
| 38 | *Maranthes glabra* | *Chrysobalanaceae* | 1.59 | 0.59 | 87.98 |
| 39 | *Erythrophleum suaveolens* | *Fabaceae* | 1.54 | 0.58 | 88.56 |
| 40 | *Macaranga barteri* | *Euphorbiaceae* | 1.51 | 0.56 | 89.12 |
| 41 | *Irvingia gabonensis* | *Irvingiaceae* | 1.44 | 0.54 | 89.66 |
| 42 | *Ganophyllum giganteum* | *Sapindaceae* | 1.43 | 0.54 | 90.20 |
| 43 | *Diospyros ferrea* | *Ebenaceae* | 1.40 | 0.53 | 90.72 |
| 44 | *Isolona hexaloba* | *Annonaceae* | 1.31 | 0.49 | 91.22 |
| 45 | *Omphalocarpum elatum* | *Sapotaceae* | 1.31 | 0.49 | 91.71 |
| 46 | *Oncoba mannii* | *Salicaceae* | 1.31 | 0.49 | 92.20 |
| 47 | *Cola griseiflora* | *Malvaceae* | 1.24 | 0.46 | 92.66 |
| 48 | *Pancovia laurentii* | *Sapindaceae* | 1.22 | 0.46 | 93.12 |
| 49 | *Alstonia congensis* | *Apocynaceae* | 1.20 | 0.45 | 93.57 |
| 50 | *Xylopia hypolampra* | *Annonaceae* | 1.06 | 0.40 | 93.97 |
| 51 🡪 117 | *Rest* | *Rest* | - | - | 100 |

**S2 File. Table C. Kenward-Rogers approximation. Result of the analysis of variance with Kenward-Roger approximation for degrees of freedom. The results show the fixed parameters selected are all significant.**

|  | Sum squared | Mean square | F value | P-value(>F) |
| --- | --- | --- | --- | --- |
| Regeneration guild | 0.0385 | 0.01926 | 8.32 | < 0.01 |
| Distance to the pith | 0.0114 | 0.01137 | 4.91 | <0.05 |
| Shade-tolerant * Distance to the pith | 3.3213 | 1.66064 | 717.56 | <0.001 |
